# Co-localization of major quantitative trait loci for pod size and weight to a 3.7 cM interval on chromosome A05 in cultivated peanut (*Arachis hypogaea* L.)

**DOI:** 10.1186/s12864-016-3456-x

**Published:** 2017-01-09

**Authors:** Huaiyong Luo, Xiaoping Ren, Zhendong Li, Zhijun Xu, Xinping Li, Li Huang, Xiaojing Zhou, Yuning Chen, Weigang Chen, Yong Lei, Boshou Liao, Manish K. Pandey, Rajeev K. Varshney, Baozhu Guo, Xiangguo Jiang, Fei Liu, Huifang Jiang

**Affiliations:** 1 Key Laboratory of Biology and Genetic Improvement of Oil Crops, Ministry of Agriculture, Oil Crops Research Institute of the Chinese Academy of Agricultural Sciences, Wuhan, 430062 China; 2 International Crops Research Institute for the Semi-Arid Tropics (ICRISAT), Hyderabad, 502324 India; 3 Crop Protection and Management Research Unit, USDA-ARS, Tifton, GA 31793 USA; 4 Xiangyang Academy of Agricultural Sciences, Xiangyang, 461057 China

**Keywords:** Peanut, QTL, Pod length, Pod width, Hundred-pod weight, Yield

## Abstract

**Background:**

Cultivated peanut (*Arachis hypogaea* L.), an important source of edible oil and protein, is widely grown in tropical and subtropical areas of the world. Genetic improvement of yield-related traits is essential for improving yield potential of new peanut varieties. Genomics-assisted breeding (GAB) can accelerate the process of genetic improvement but requires linked markers for the traits of interest. In this context, we developed a recombinant inbred line (RIL) mapping population (Yuanza 9102 × Xuzhou 68-4) with 195 individuals and used to map quantitative trait loci (QTLs) associated with three important pod features, namely pod length, pod width and hundred-pod weight.

**Results:**

QTL analysis using the phenotyping data generated across four environments in two locations and genotyping data on 743 mapped loci identified 15 QTLs for pod length, 11 QTLs for pod width and 16 QTLs for hundred-pod weight. The phenotypic variation explained (PVE) ranged from 3.68 to 27.84%. Thirteen QTLs were consistently detected in at least two environments and three QTLs (*qPLA05.7*, *qPLA09.3* and *qHPWA05.6*) were detected in all four environments indicating their consistent and stable expression. Three major QTLs, detected in at least three environments, were found to be co-localized to a 3.7 cM interval on chromosome A05, and they were *qPLA05.7* for pod length (16.89–27.84% PVE), *qPWA05.5* for pod width (13.73–14.12% PVE), and *qHPWA05.6* for hundred-pod weight (13.75–26.82% PVE). This 3.7 cM linkage interval corresponds to ~2.47 Mb genomic region of the pseudomolecule A05 of *A. duranensis*, including 114 annotated genes related to catalytic activity and metabolic process.

**Conclusions:**

This study identified three major consistent and stable QTLs for pod size and weight which were co-localized in a 3.7 cM interval on chromosome A05. These QTL regions not only offer further investigation for gene discovery and development of functional markers but also provide opportunity for deployment of these QTLs in GAB for improving yield in peanut.

**Electronic supplementary material:**

The online version of this article (doi:10.1186/s12864-016-3456-x) contains supplementary material, which is available to authorized users.

## Background

Cultivated peanut or groundnut (*Arachis hypogaea* L.) is an allotetraploid (2n = 4x = 40) legume crop and is widely grown worldwide in >100 countries with global annual production of 42.32 million tonnes (FAOSTAT, 2014). Peanut is an important oil crop and has a key role in human nutrition [1]. Improving yield has been one of the major objectives in peanut breeding programs, which is directly influenced by pod-related traits (PRTs) [2–4]. Quantitative traits, including PRTs, show complex interaction with environment leading to varied productivity under different environments. In order to select a promising line for varietal release, breeders need to assess its potential in multiple environments to check its stable performance to achieve higher adoption in the farmers’ field. In a breeding program, it is very difficult and expensive to screen large number of lines across multiple environments for yield assessment. Genomics-assisted breeding (GAB) has potential to accelerate the process of achieving higher genetic gain in less time and with minimum resources using molecular markers [4, 5]. In order to deploy GAB, linked markers for PRTs is essential for developing high yielding peanut varieties.

Quantitative trait locus (QTL) mapping using bi-parental population has been widely conducted successfully to identify the genomic regions associated with quantitative traits in several crop plants [6, 7] including peanut. In recent years, QTLs associated with economically important traits such as disease resistance [8, 9], drought tolerance [10, 11], seed and oil quality [12, 13], agronomic and yield traits [14, 15] were identified in peanut crop. Molecular markers tightly linked to QTLs after validation can be further deployed in GAB [5, 16]. For example, one major QTL for rust resistance was introgressed from resistant cultivar ‘GPBD 4’ into three early maturing elite varieties through marker-assisted backcrossing (MABC) [17].

Limited efforts were made in identifying QTLs controlling PRTs in peanut which did not provided significant results deployable in breeding program. For example, Selvaraj et al. [4] identified two SSR markers, PM375 and Seq8D09, linked with pod length using bulked segregant analysis. Similarly, Shirasawa et al. [18] identified three QTLs for pod length and two for pod width in an F_2_ population while Fonceka et al. [19] mapped three QTLs for pod length, six for pod width and two for hundred-pod weight in an advanced backcross population. More recently, Huang et al. [15] detected one QTL for pod length, two QTLs for pod width and three QTLs for hundred-pod weight in an F_2:3_ population. In addition to above, Chen et al. [3] detected 22 QTLs for pod length and width in two F_2:3_ populations. However, quantitative traits are highly influenced by environments and QTLs identified at one specific location may not be valid for another location with varied environmental conditions [14]. Majority of the studies identified QTLs in segregating populations and not in fixed population such as RIL population.

The RIL population can be repeatedly used for generation of phenotyping data in multiple environments which is a key factor in doing genetic dissection of complex and quantitative traits, thereby helping in precise identification of consistent and stable QTLs. The variety Yuanza 9102 is small-podded with low pod weight while the variety Xuzhou 68-4 has large pods and higher pod weight. In this study, a RIL population was developed from the cross between Yuanza 9102 and Xuzhou 68-4 and used to identify QTLs controlling yield-related traits such as pod length (PL), pod width (PW), and hundred-pod weight (HPW) across four environments.

## Methods

### Plant materials

A recombinant inbred line (RIL) population in F_5_ generation was developed from a cross between Yuanza 9102 and Xuzhou 68-4 using single seed decent method to construct a dense genetic linkage map and conducting QTL analysis for pod features. The female parent, Yuanza 9102, belongs to *A. hypogaea* subsp. *hypogaea* var. *vulgaris* and is derived from interspecific hybridization between the cultivated peanut Baisha1016 and wild species *A. chacoense*. The male parent, Xuzhou 68-4, belongs to *A. hypogaea* subsp. *hypogaea* var. *hypogaea* and has significantly larger pods than the female parent, Yuanza 9102. A total of 195 recombinant inbred lines (RILs) were used in the present study for generating genotyping and phenotyping data followed by genetic map construction and QTL analysis.

### Field trials for generating phenotyping data

Phenotyping data was generated on the RIL population for four environments i.e., three environments at Wuhan (WH), China (F_5_ generation during 2013, F_6_ generation during 2014 and F_7_ generation during 2015) while single environment at Xiangyang (XY), China (F_7_ generation during 2015). These experiments were designated as WH2013, WH2014, WH2015 and XY2015, respectively. Each environment was a field trial conducted at a location in a year in this paper. The random block design (RBD) with three replications was adopted for generating phenotyping data during all the four environments. Each RIL was planted in a 2.5 m long single-row and row-to-row space was 33 cm. There were 12 plants in each row with plant-to-plant distance of 20 cm. Of these 12 plants, 8 plants in the middle of each row were harvested for trait measurement. Three important pod related traits (PRTs), pod length (PL), pod width (PW) and hundred-pod weight (HPW), were measured three times for each replication according to previously described standard procedures [15, 20]. To reduce the influence of environmental factors, the mean trait value in each trial was used in analysis.

### Statistical analysis of phenotyping data

Statistical analysis for the phenotypic data of PRTs was conducted using IBM SPSS Statistics Version 22 software [21]. The Shapiro-Wilk (w) statistic was used to test the null hypothesis that the phenotypic data were normally distributed. The univariate variance analyses were performed using standard GLM method and variance components were estimated using restricted maximum likelihood (REML) method. The broad-sense heritability for each trait across the four environment trials was calculated based on the estimated variance components with the following formula: ${H}^2={\upsigma}_g^2/\left({\upsigma}_g^2+{\upsigma}_{g\times e}^2+{\upsigma}_e^2\right)$ based on plot mean and ${H}^2={\upsigma}_g^2/\left({\upsigma}_g^2+{\upsigma}_{g\times e}^2/r+{\upsigma}_e^2/rn\right)$ based on entry mean, where ${\upsigma}_g^2$ is the genotypic variance component among RILs, ${\upsigma}_{g\times e}^2$ is the RILs × environment interaction variance component, ${\upsigma}_e^2$ is the residual (error) variance component, and r is the number of environment trials, n is the number of replications in each field experiment [22]. Correlation coefficients between each pair of the three traits were also calculated using IBM SPSS Statistics Version 22 software [21].

### Genotyping of mapping population

A total of 8,112 SSR markers from either published reports [18, 23–38] or newly developed SSR markers (unpublished) from the genome sequences of diploid ancestors [1] were used to screen the polymorphism between parental genotypes of the RIL population. Polymorphic markers were used to genotype complete RIL population along with parental genotypes. Genomic DNA was extracted from young leaves collected from RILs in F_5_ generation using a modified CTAB method [39]. The integrity and quality of the DNA was evaluated on a 1% agarose gel by comparison with uncut lambda DNA. PCR amplification was conducted in a 10 μl volume, containing 20 ng DNA template, 0.5 μM each primer, 1× PCR buffer, 1 mM MgCl_2_, 0.2 mM dNTP and 0.5 U Taq polymerase. PCR was performed with a Bio-Rad T100 Thermal Cycler using the standard PCR program with little modification i.e., 95 °C for 4 min; 35 cycles of 94 °C for 55 s, 55–58 °C (varies for each primer pair) for 45 s, and 72 °C for 1 min; and a final extension step of 72 °C for 10 min. The PCR products were separated on a 6% polyacrylamide gel and visualized by silver staining [40].

### Construction of genetic linkage map

Pearson’s Chi square test was used to assess the goodness of fit to the expected segregation ratio 15:2:15 for co-dominant marker or 17:15 for dominant marker (*P* < 0.05). A genetic linkage map was constructed using the JoinMap 4.0 [41] with a maximum recombinant frequency of 0.4. The recombination ratio was converted to genetic distance by the Kosambi mapping function [42]. The linkage groups (LGs) were designated as chromosome A01-A10 and B01-B10 based on the common markers as a previously published integrated consensus map [43]. This consensus map was constructed based on 16 genetic linkage maps [43] and used as reference in other publications [3, 15, 44]. The graphical presentation of genetic linkage map was generated with the MapChart 2.3 software [45].

### QTL analysis

Genome-wide QTL mapping was performed using the mean value of each trait in each environment. QTL analysis was conducted using the composite interval mapping (CIM) method [46] in the Windows QTL Cartographer 2.5 software [47]. The standard CIM model (model 6) and forward regression method were selected. The number of control markers, window size and walk speed were 5, 10 and 2 cM, respectively. The threshold of LOD for declaring the presence of a QTL was determined by 1000 permutation tests. When separated by a minimum distance of 20 cM, two peaks on one chromosome were considered as two different QTLs [10]. Otherwise, the higher peak was chosen to more closely approximate the position of the QTL. If QTLs for the same trait detected in different environments had overlapping 2-LOD support intervals, they were considered to be the same QTL and also been designated as consistent QTLs. Similarly, if the same QTL appeared in both the locations (Wuhan and Xiangyang), such QTLs were refereed as stable QTLs. QTLs were designated with an initial letter ‘q’ followed by the trait name and the LG corresponding chromosome, similar to the previously described nomenclature [48]. After the linkage group, a number was added if more than one QTL was detected for the same trait and linkage group. For example, if two QTLs for pod length were detected on chromosome A05, they were named as *qPLA05.1* and *qPLA05.2*, respectively. If QTLs for different traits had overlapping 2-LOD support intervals, they were clustered in specific co-localized chromosomal regions. Genome sequences and annotations of the diploid ancestors of cultivated peanut were downloaded from PeanutBase [1]. Molecular markers were positioned on the chromosomal pseudomolecules using BLAST and ePCR (electronic PCR) with high similarity parameters (taking the top hits only, with placement by BLAST ($\mathrm{e}\;\mathrm{value}<1\times {10}^{-10}$) given preference over ePCR where both were available) [1].

## Results

### Phenotypic variation of Pod Related Traits (PRTs)

**Fig. 1:**
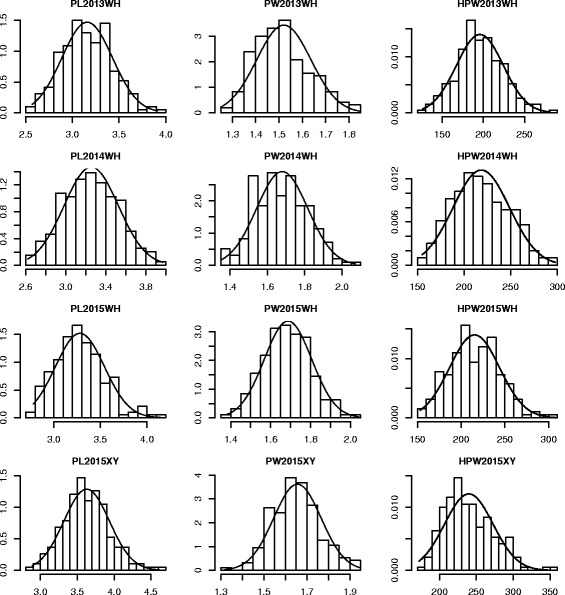
Phenotype distribution of pod length, pod width and hundred-pod weight. The *y-axis* represented density, while the *x-axis* represented values of each trait. The normal distribution curve in each graph represented the expected density. WH Wuhan, XY Xiangyang, PL Pod lentth, PW pod width, HPW hundred-pod weight. PL2013WH means pod length in Wuhan 2013, etc

Significant differences were found between the two parents for various PRTs across four environments i.e., WH2013, WH2014, WH2015 and XY2015 (Table 1). Large phenotypic variations for the PRTs were observed among RILs in all the four environments, showing continuous distributions with transgressive segregation (Table 1, Fig. 1). The normality test indicated that the phenotypic data were normally distributed for PRTs, except pod weight (PW) in WH2013 trial, pod length (PL) in WH2015 trial and hundred-pod weight (HPW) in XY2015 trial (Table 1, Fig. 1). Variance analysis for the PRTs across the four trials showed significant differences among RILs, environments and RILs × environment interactions (Table 2). The values of broad sense heritability were estimated to be 0.70 for pod length, 0.51 for pod weight and 0.66 for hundred-pod weight based on plot mean while these estimates were much higher based on entry mean, such as 0.92 for pod length, 0.83 for pod weight and 0.90 for hundred-pod weight. Correlation analysis indicated that the three PRTs had significant positive association between each other (Table 3) and therefore positive relationship with potential yield.

**Table 1:** Descriptive statistical analysis of phenotypes of pod-related traits in the RIL population

Env	Trait	P1	P2	Range	Mean	SD	Skew	Kurt	w(Sig)
WH2013	PL(cm)	3.07	3.59	2.57–3.91	3.16	0.27	0.24	−0.25	0.99(0.304)
	PW(cm)	1.49	1.71	1.25–1.85	1.52	0.12	0.47	−0.23	0.97(0.003)
	HPW(g)	178.31	222.26	126.6–281.52	195.84	28.46	0.19	0.01	0.99(0.891)
WH2014	PL(cm)	2.90	3.70	2.62–3.97	3.26	0.27	0.09	−0.46	0.99(0.582)
	PW(cm)	1.58	1.87	1.35–2.07	1.68	0.14	0.13	−0.31	0.99(0.635)
	HPW(g)	190.06	273.19	155.23–297.5	218.45	30.30	0.23	−0.59	0.99(0.066)
WH2015	PL(cm)	2.87	3.55	2.78–4.18	3.28	0.26	0.52	0.33	0.98(0.009)
	PW(cm)	1.60	1.78	1.38–2.04	1.69	0.12	0.12	−0.02	0.99(0.926)
	HPW(g)	179.90	267.33	153–306.94	215.30	28.55	0.22	−0.10	0.99(0.410)
XY2015	PL(cm)	3.61	4.07	2.89–4.61	3.63	0.31	0.32	0.35	0.99(0.289)
	PW(cm)	1.49	1.73	1.32–1.95	1.66	0.11	0.14	0.00	0.99(0.641)
	HPW(g)	195.63	248.84	172.13–354.7	239.75	32.84	0.63	0.23	0.97(0.000)

*Env* Environment, *P1* female parent Yuanza 9102, *P2* male parent Xuzhou 68-4, *SD* standard deviation, *Skew* Skewness, *Kurt* Kurtosis, *w* Shariro-Wilk statistic value, *Sig* Significance, *WH* Wuhan, *XY* Xiangyang, *PL* Pod length, *PW* pod width, *HPW* hundred-pod weight

**Table 2:** Analysis of variance for pod-related traits in the RIL population across four environment trials

Trait	Variables	df	Mean square	F-value	*P*-value
PL	RILs	194	0.498	49.257	<0.001
	Evironments	3	16.382	1620.365	<0.001
	RILs × Evironments	572	0.040	3.933	<0.001
	Error	768	0.010		
PW	RILs	194	0.077	26.852	<0.001
	Evironments	3	3.323	808.951	<0.001
	RILs × Evironments	572	0.013	4.499	<0.001
	Error	768	0.003		
HPW	RILs	194	5470.988	70.586	<0.001
	Evironments	3	125432.506	1618.314	<0.001
	RILs × Evironments	572	567.502	7.322	<0.001
	Error	768	77.508		

*PL* Pod length, *PW* pod width, *HPW* hundred-pod weight

**Table 3:** Correlation analysis for pod-related traits in the RIL population

Env	Trait	PL	PW	HPW
WH2013	PL	1		
	PW	0.725^a^	1	
	HPW	0.738^a^	0.669^a^	1
WH2014	PL	1		
	PW	0.736^a^	1	
	HPW	0.789^a^	0.910^a^	1
WH2015	PL	1		
	PW	0.610^a^	1	
	HPW	0.814^a^	0.805^a^	1
XY2015	PL	1		
	PW	0.489^a^	1	
	HPW	0.674^a^	0.635^a^	1

^a^Correlation is significant at the 0.01 level

*WH* Wuhan, *XY* Xiangyang, *PL* pod length, *PW* pod width, *HPW* hundred-pod weight

### Molecular marker polymorphisms and genetic map construction

Out of 8,112 SSR markers screened on the parental genotypes of the RIL population, 729 markers showed polymorphisms in the parents as well as in the RIL population (Additional file 1: Table S1). Among them, one marker AHGS0729 amplified three genetic loci and 12 markers amplified two loci, while the remaining 716 markers amplified a single locus. Among these 743 genetic loci, 660 loci were co-dominant and 83 loci were dominant. The Chi square analysis identified 356 loci (47.91%) with segregation distortion. A genetic linkage map containing 743 loci was constructed spanning 1,232.57 cM with an average inter-marker distance of 1.66 cM (Table 4, Additional file 2: Table S2). All the 743 loci were assigned to 22 LGs whose length varied from 9.47 cM to 119.48 cM and number of mapped loci ranged from 3 to 97 marker loci (Table 4, Additional file 3: Figure S1). Based on 292 common markers which were also included in a previously published integrated consensus map [43], 19 of the 22 LGs were assigned to 17 chromosomes of the A and B subgenomes (Table 4, Additional file 4: Figure S2). Chromosome A01 was found to be divided into two LGs (LG01 and LG02) due to insufficient linkage between them. Similarly, chromosome A08 was divided into LG08 and LG09.

**Table 4:** Description of the genetic linkage map constructed in this study

LGs	Chrom	Length(cM)	Loci^a^	Common loci^b^
LG01	A01	86.62	61	23
LG02	A01	39.84	6	4
LG03	A03	42.98	10	6
LG04	A04	20.47	6	2
LG05	A05	99.27	97	24
LG06	A06	55.05	26	7
LG07	A07	46.26	38	19
LG08	A08	50.86	23	12
LG09	A08	15.43	3	1
LG10	A09	86.68	56	24
LG11	A10	13.78	3	2
LG12	B01	67.91	64	33
LG13	B02	80.57	77	32
LG14	B03	72.29	7	4
LG15	B04	119.48	71	34
LG16	B05	83.97	81	33
LG17	B09	42.56	7	3
LG18	B10	63.95	85	29
LG19	-	106.19	11	0
LG20	-	16.12	5	0
LG21	-	9.47	3	0
LG22	-	12.82	3	0
Total	-	1,232.57	743	292

^a^Number of loci in each linkage group

^b^Number of common markers which were contained in a previously published integrated consensus linkage map [43]

### Detection of QTLs for Pod Related Traits (PRTs)

**Fig. 2:**
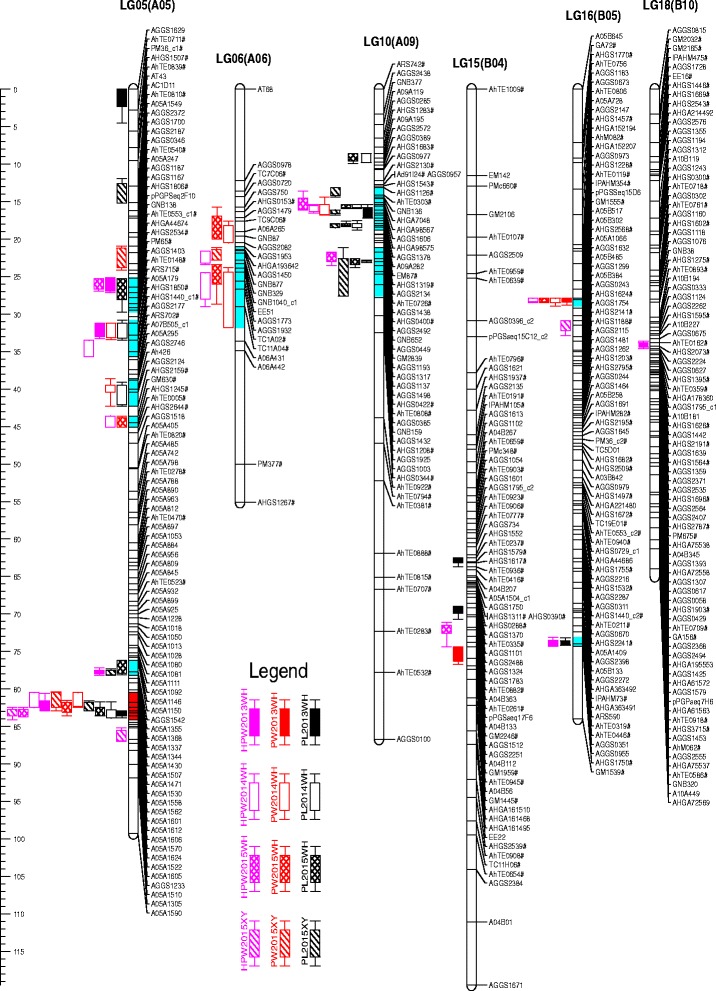
QTLs distribution of pod-related traits in the genetic map. Loci with “#” were common markers which also included in a previously published integrated consensus linkage map [43]. WH Wuhan, XY Xiangyang, PL Pod length, PW pod width, HPW hundred-pod weight. PL2013WH means QTL for pod length detected in Wuhan 2013, etc. The co-localized regions of QTLs for different traits were highlighted in *blue* or *red color* on the chromosome bars

QTL analysis using phenotyping and genotyping data identified a total of 65 QTLs with 3.68 to 27.84% phenotypic variation explained (PVE) associated with the PRTs in the four environments (Fig. 2, Additional file 5: Table S3). For pod length, six QTLs were detected in WH2013 trial (5.45–16.89% PVE), seven QTLs in WH2014 trial (5.27–27.84% PVE), seven QTLs in WH2015 trial (9.33–23.91% PVE), and seven QTLs in XY2015 trial (3.68–25.68% PVE). For pod width, two QTLs were detected in WH2013 trial (5.88–8.90% PVE), seven QTLs in WH2014 trial (5.26–14.03% PVE), five QTLs in WH2015 trial (6.42–13.73% PVE), and three QTLs in XY2015 trial (5.40–14.12% PVE). For hundred-pod weight, six QTLs were detected in WH2013 trial (4.81–21.74% PVE), seven QTLs in WH2014 trial (5.72–21.29% PVE), five QTLs in WH2015 trial (4.12–26.82%), and three QTLs in XY2015 trial (6.52–13.75% PVE).

As shown in Fig. 2, some QTLs detected in different environments for the same trait had overlapping 2-LOD support intervals, and they were considered to be one QTL which could be repeatedly detected. Therefore, the 65 loci detected in four environment trials were designated as 15 QTLs for pod length, 11 QTLs for pod width, and 16 QTLs for hundred-pod number (Additional file 5: Table S3).

For pod length, the 15 QTLs were identified on chromosomes A05, A09, B04 and B05. Two QTLs, *qPLA05.7* and *qPLA09.3*, were found to be consistent and stable as they were detected in all four environments. Flanked by the marker A05A1430 and A05A1601 on chromosome A05, the QTL for pod length, *qPLA05.7*, explained 16.89, 17.84, 23.91 and 25.68% of the phenotypic variance in WH2013, WH2014, WH2015 and XY2015 environments, respectively. Similarly, another QTL for pod length, *qPLA09.3*, flanked by AGGS1606 and AGGS2134 on chromosome A09 explained 5.45, 17.76, 14.47 and 12.41% of the phenotypic variance in four environments, respectively. Further, two additional QTLs for pod length, *qPLA09.4* (AGGS2134 - AGGS2492) and *qPLA09.5* (AGGS1137 - AGGS1925), were mapped on chromosome A09 in three environments (WH2014, WH2015 and XY2015) with 12.05–16.91% and 11.33–16.86% PVE, respectively.

For pod width, of the 11 QTLs identified on five chromosomes (A05, A06, A09, B04 and B05), two QTLs, *qPWA05.5* and *qPWB05*, were consistent and stable in expression as they were detected in three environments (WH2014, WH2015 and XY2015). The first QTL *qPWA05.5* (A05A1344 - A05A1562) had showed 13.73–14.12% PVE while the second QTL *qPWB05* (AHGA152207 - AHGS1228) showed 5.88–7.30% PVE in WH2014, WH2015 and XY2015 trials.

Similarly for hundred-pod weight, the 16 QTLs were identified on chromosomes A05, A06, A09, B04, B05 and B10. A major QTL on chromosome A05, designated as *qHPWA05.6*, was detected in all four environments and hence consistent and stable. Interestingly, it was flanked by the same markers (A05A1430 - A05A1601) as the major QTL *qPLA05.7* for pod length and had shown 21.74, 21.29, 26.82 and 13.75% PVE in WH2013, WH2014, WH2015 and XY2015, respectively.

### A co-localized region of stable and major QTLs for PRTs on chromosome A05

**Fig. 3:**
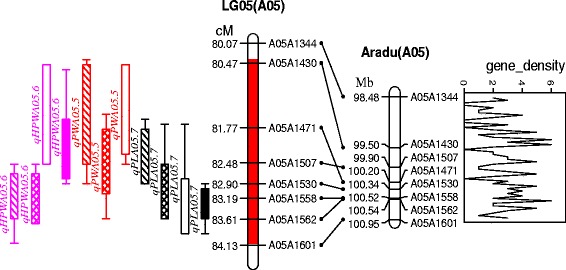
Genetic and physical maps of the dominant and co-localized interval on chromosome A05. The legend for QTLs was the same as that in Fig. 2. Gene density indicated gene numbers per 50 kb interval

A total of 11 chromosomal regions harbored QTLs for different traits where multiple QTLs were mapped (Fig. 2). This phenomenon was not unexpected given the strong positive correlations among the three traits (Table 3), indicating the existing of pleiotropic effects of single gene or tight linkage. A co-localized QTL interval close to the end region of chromosome A05 was significantly more dominant than others. It was located at 80.4–84.1 cM map position on chromosome A05 and covering around 3.7 cM in length with flanking markers A05A1344 and A05A1601 (Figs. 2 and 3). This region harbored the major QTLs for pod length (*qPLA05.7*), pod width (*qPWA05.5*), and hundred-pod weight (*qHPWA05.6*) (Fig. 3, Table 5)*.* Each QTL was detected at least in three environments and hence more consistent and stable in expression.

**Table 5:** QTLs harbored in the dominant and co-localized interval on chromosome A05

Trait	QTL	Marker interval	Location & year	LOD	Additive	PVE(%)
PL	*qPLA05.7*	A05A1430-A05A1601	Wuhan 2013	11.1	0.1154	16.89
			Wuhan 2014	22.7	0.1490	27.84
			Wuhan 2015	19.4	0.1297	23.91
			Xiangyang 2015	18.6	0.1595	25.68
PW	*qPWA05.5*	A05A1344-A05A1562	Wuhan 2014	9.9	0.0541	14.03
			Wuhan 2015	9.0	0.0538	13.73
			Xiangyang 2015	8.0	0.0441	14.12
HPW	*qHPWA05.6*	A05A1430-A05A1601	Wuhan 2013	13.0	13.6342	21.74
			Wuhan 2014	14.6	14.6633	21.29
			Wuhan 2015	16.3	15.6463	26.82
			Xiangyang 2015	7.5	12.8723	13.75

*PL* pod length, *PW* pod width, *HPW* hundred-pod weight, *PVE* phenotypic variation explained

BLAST searching and ePCR of eight markers mapped in this region could be traced to the pseudomolecule A05 of A subgenome (*A. duranensis* V14167) [1] (Fig. 3). The corresponding position of 3.7 cM on the genetic map was about 2.47 Mb in the physical map i.e., 98,478,303 bp to 100,945,376 bp containing 114 putative genes [1]. Fifteen novel genes encoded unknown proteins, while the other 99 genes had reported homologs (Additional file 6: Table S4). Protein Aradu.H6FXW might have a function in the regulation of cell proliferation. Eight genes might encode transcription factors, including homeobox transcription factor (Aradu.5W2E7, Aradu.WUY99 and Aradu.S4P4Y), transcription factor IIS (Aradu.C2I2I and Aradu.TWC9B), MYB transcription factor (Aradu.BVL00) and others (Aradu.C989A and Aradu.PSF4U). As shown in Additional file 6: Table S4, 56 of the 99 genes were assigned at least one GO term. Among the various biological processes, metabolic process (41.1%) and cellular process (21.4%) were most highly represented (Additional file 7: Figure S3). The genes involved in other important biological processes such as biological regulation, cellular component organization, establishment of localization, pigmentation and response to stimulus, were alos identified through GO annotations. Similarly, binding (57.1%) and catalytic activity (46.4%) were most represented among the various molecular functions, and cell (26.8%) and cell part (26.8%) were most represented among the cellular components (Additional file 7: Figure S3). Enrichment analysis indicated that 30 of the 56 genes were enriched in 48 GO terms using all gene models of A subgenome assembly (*A. duranensis* V14167) as reference (*P* < 0.05), including carbon-oxygen lyase activity, pectate lyase activity and other catalytic activities (Additional file 8: Table S5). Through KEGG analyses, a total of 11 genes encoding oxidase, dehydrogenase, lyase, synthase, dehydratase and lactoperoxidase were assigned to 16 biological pathways, including amino acid metabolism, carbohydrate metabolism, energy metabolism, metabolism of cofactors and vitamins and biosynthesis of antibiotics and other secondary metabolites (Additional file 9: Table S6).

## Discussion

The broad-sense heritability estimated in this study was relatively high for pod length, pod width and hundred-pod weight, indicating that genetic factors play a major role in determination of these traits, although influenced by environment. In this study, a RIL population was used to construct a dense genetic linkage map and conducting QTL analysis for pod features. A genetic linkage map is a prerequisite to efficiently identify molecular markers associated with quantitative traits. Because of a lack of polymorphism at the DNA level, the first SSR-based genetic linkage map for peanut only had 135 SSR loci. In this study, a genetic linkage map containing 743 loci was constructed using JoinMap 4.0 [41]. It is an user-friendly and widely used commercial software in the scientific community [49], although it was outperformed by some recent tools [49, 50] at speed or manipulation of noisy data. The constructed linkage map covered a total length of 1,232.57 cM an average inter-marker distance of 1.66 cM. The loci number and density of our map were relatively higher than that of recent studies [3, 10, 15, 51], except for the integrated consensus map [43]. Using this linkage map, fifteen QTLs were identified for pod length, 11 QTLs for pod width and 16 QTLs for hundred-pod weight (Additional file 5: Table S3) in the RIL population across four environments. The LOD values of these QTLs ranged from 3.2 to 22.7 and were higher than the threshold of LOD for declaring the presence of a QTL which was determined by 1000 permutation tests. All the linked markers identified for pod related traits after validation can be deployed in breeding for marker-based selection to improve yield in peanut.

### QTLs for pod related traits with stable performance

Besides identification of QTLs, it is very important to assess their stable performance across varied environments. A similar study conducted by Chen et al. [3] detected six QTLs for pod length and eight QTLs for pod width in a F_2:3_ populations in two environments, but none of them were detected in both environments. Despite the significant G × E interactions (*P* < 0.001) present in the four trials conducted in this study, three major QTLs (*qHPWA05.6* for hundred-pod weight, *qPLA05.7* and *qPLA09.3* for pod length) have shown stable performance across four environments and two locations. In addition, four QTLs for pod length, four QTLs for pod width and two QTLs for HPW were detected in two or three trials. Such QTLs with stable performance for pod related traits have been identified for the first time in peanut and will be very useful for further fine mapping of the QTL region and development of diagnostic markers to use in breeding.

The present study reports 15 QTLs for pod length mapped on chromosomes A05, A09, B04 and B05, the three QTLs (*qPLB04.1*, *qPLB04.2* and *qPLB05*) were not detected in earlier studies, hence, novel QTLs. The chromosomes A05 and A09 might harbor important genes for pod length as seven QTLs from this study and six QTLs from earlier studies [3, 18] were mapped on A05, and five QTLs from this study and six QTLs from previous studies [3, 4, 18, 19] were identified on A09. Of these QTLs, two QTLs, *qPLA05.7* and *qPLA09.3*, identified in this study had stable expression across environments.

Similarly, of the 11 QTLs identified for pod width in this study on chromosomes A05, A06, A09, B04 and B05, QTLs identified on chromosomes A06 (*qPWA06.1*, *qPWA06.2*, *qPWA06.3)* and B04 (*qPWB04)* were novel QTLs. Chromosome A05 seems very important and might harbor important genes for pod width, as five QTLs from present study and six QTLs from previous studies [3, 15] were mapped on this chromosome. The QTL *qPWA05.5* explained the largest phenotypic variations and consistently expressed across environments. The two QTLs reported by previous study conducted by Chen et al. [3] and one QTL, *qPWA09*, identified in this study were mapped on the chromosome A09. Similarly, two QTLs identified by Fonceka et al. [19] and one QTL, *qPWB05*, detected in this study were mapped on the chromosome B05.

The 16 QTLs detected in the present study for hundred-pod weight were mapped on the chromosomes A05, A06, A09, B04, B05 and B10, while the five QTLs reported in previous studies [15, 19] were located on chromosomes A07, B02, B03 and B05. Therefore, the 16 QTLs identified in the present study were novel in nature. Of these QTLs, the QTL *qHPWA05.6* was the most consistent and stable one. The above results suggested that these pod-related traits are quantitative in nature controlled by multiple genomic regions and their effects were often affected by the environment.

### Co-localized region on chromosome A05 play a major role in controlling pod related traits

The present study identified a co-localized genomic region on A05 harboring QTLs for pod related traits. This region harbored one important QTL for each pod related traits i.e., *qPLA05.7* for pod length, *qPWA05.5* for pod width, and *qHPWA05.6* for hundred-pod weight. This region also provided a significant level of contribution to phenotypic variation explained by these QTLs i.e., 16.89–27.84% PVE for pod length and 13.75–26.82% PVE for hundred-pod weight across all the four environments, and 13.73–14.12% PVE for pod width in three of the four environments. The above results indicate importance of this co-localized region for improving pod related traits through GAB. Further, this important genomic region also provides opportunity for fine mapping and development of diagnostic markers for use in improving these traits.

In addition to above mentioned further possible studies, the recently completed genome sequences of the diploid ancestors of cultivated peanut [1] provides a physical map of the highest resolution and allows the possibility to examine the co-localized region at the end of chromosome A05. The 3.7 cM genetic map distance was corresponding to the 2.47 Mb physical map region which houses 114 candidate genes. Thirteen percent of these genes are novel genes with unknown function and seems to be an enrichment of genes involved in catalytic activity and metabolic process. Eight genes were transcription factors and protein Aradu.H6FXW seems to have a function in the regulation of cell proliferation. The application of the genome sequences of wild peanut provided us an overview of candidate genes in the chromosome region of interest; however, these genes remain candidates until shown to be causally associated with the phenotypic variations in further studies.

## Conclusions

The present study identified 15 QTLs for pod length, 11 QTLs for pod width and 16 QTLs for hundred-pod weight using a RIL population across four environments in two locations. Multiple stable and major QTLs for pod related traits were co-located at the end of chromosome A05. These QTLs needs further investigation to fine map and develop diagnostic markers for these traits to use them in routine breeding program using GAB in peanut.

## Additional files

Additional file 1: Table S1. Detailed information of SSR markers used for genotyping the RIL population. (XLSX 49 kb)
Additional file 2: Table S2. Genetic linkage map constructed based on 743 polymorphic loci in the RIL population. (XLSX 28 kb)
Additional file 3: Figure S1. Graphical presentation of genetic linkage map constructed in this study. (PDF 207 kb)
Additional file 4: Figure S2. Common markers between the newly constructed linkage map and the previously published integrated consensus map. (PDF 321 kb)
Additional file 5: Table S3. QTLs detected for pod-related traits in four environments. (XLSX 15 kb)
Additional file 6: Table S4. Functional annotations of putative genes in the dominant and co-localized interval on chromosome A05. (XLSX 19 kb)
Additional file 7: Figure S3. GO annotations of genes in the dominant and co-localized interval on chromosome A05. (TIF 597 kb)
Additional file 8: Table S5. Enriched GO terms for putative genes in the dominant and co-localized interval on chromosome A05. (XLSX 29 kb)
Additional file 9: Table S6. KEGG pathways for putative genes in the dominant and co-localized interval on chromosome A05. (XLSX 12 kb)

## Abbreviations

CIM: Composite interval mapping
HPW: Hundred-pod weight
LGs: Linkage groups
PL: Pod length
PRTs: Pod-related traits
PVE: Phenotypic variance explained
PW: Pod width
QTLs: Quantitative trait loci
RILs: Recombinant inbred lines
WH: Wuhan
XY: Xiangyang
