# Supplementary figures and images for: Co-localization of major quantitative trait loci for pod size and weight to a 3.7 cM interval on chromosome A05 in cultivated peanut (Arachis hypogaea L.)
Source: BMC Genomics. 2017 Jan 9;18:58. doi: 10.1186/s12864-016-3456-x (PMC5223410; doi:10.1186/s12864-016-3456-x)

LG01(A01)

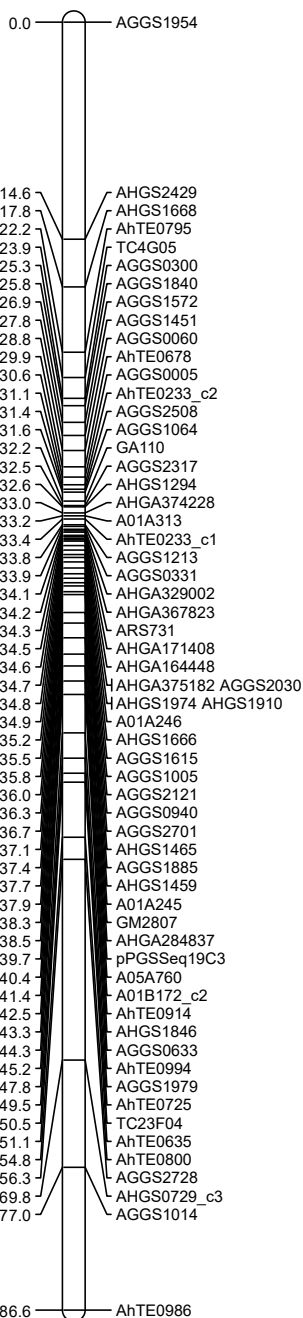

LG02(A01)

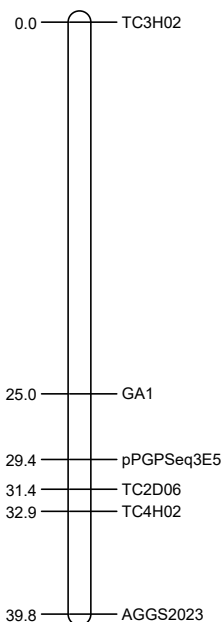

LG03(A03)

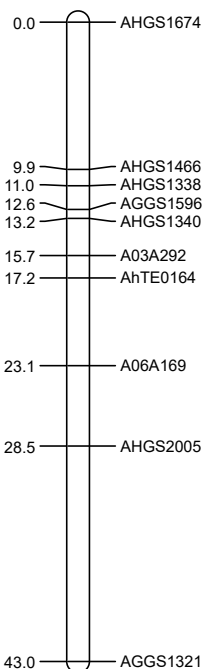

LG04(A04)

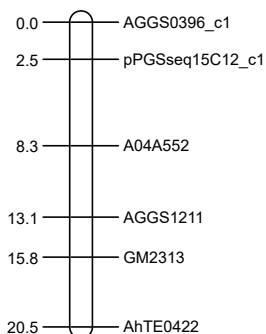

LG05(A05)

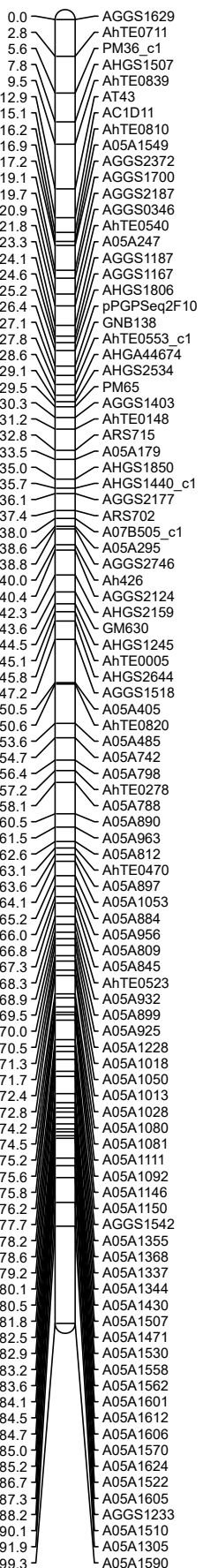

LG06(A06)

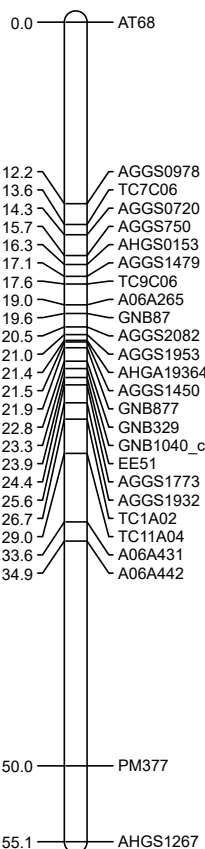

LG07(A07)

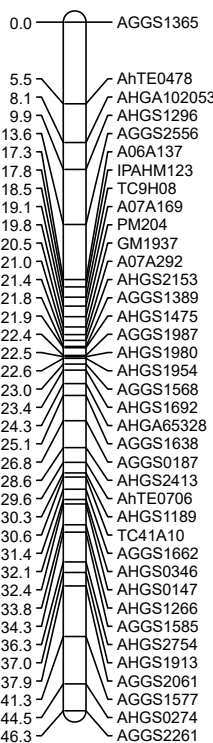

LG08(A08)

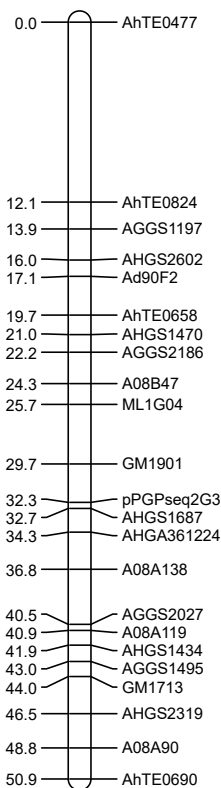

# LG09(A08)

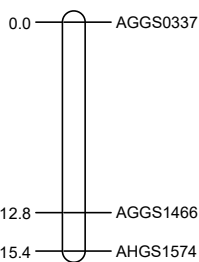

# LG10(A09)

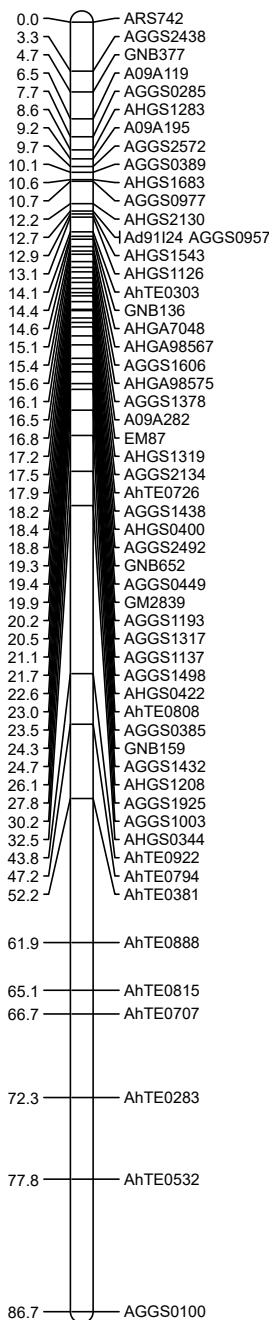

# LG11(A10)

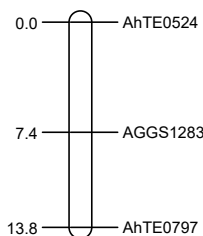

# LG12(B01)

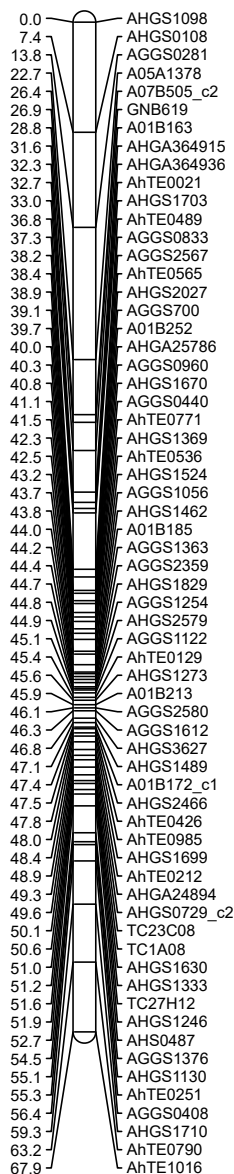

LG13(B02)

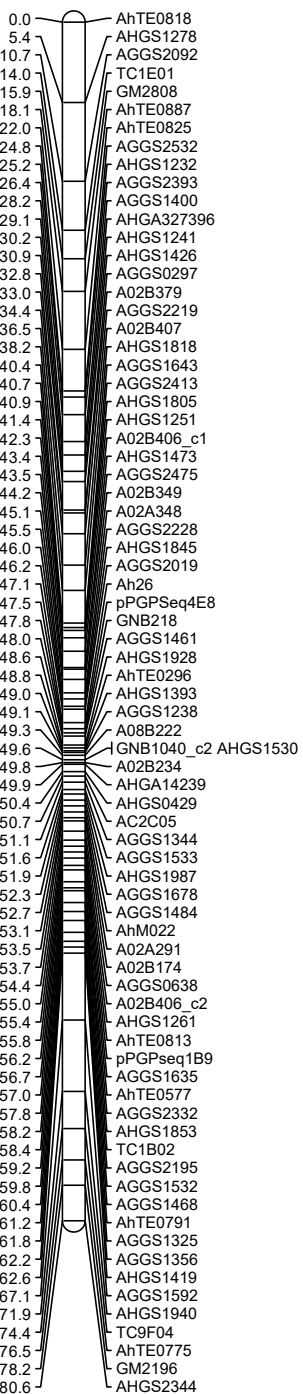

LG14(B03)

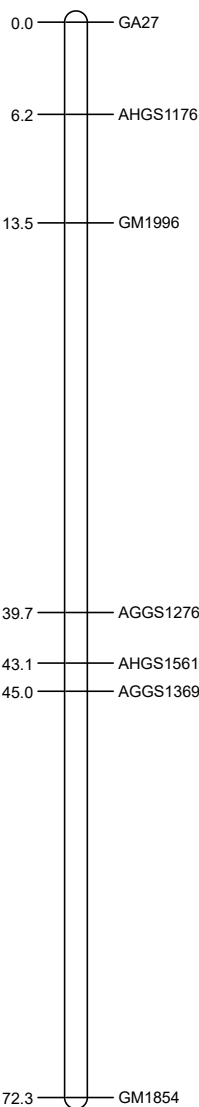

LG15(B04)

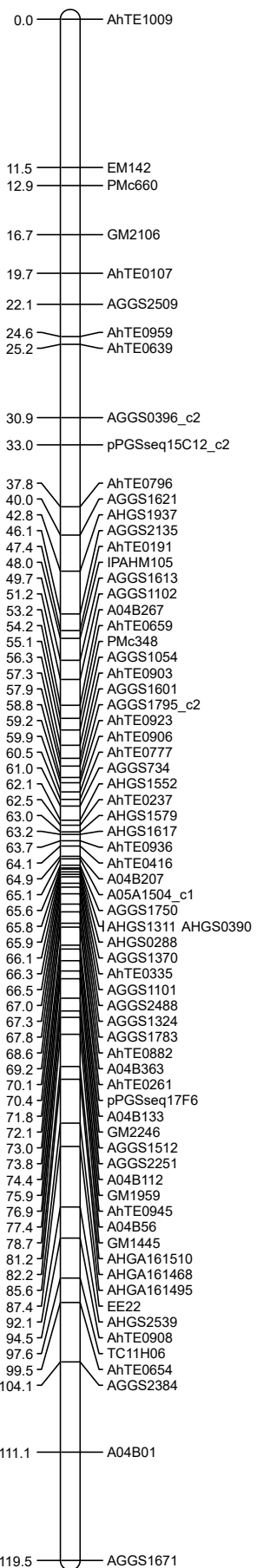

LG16(B05)

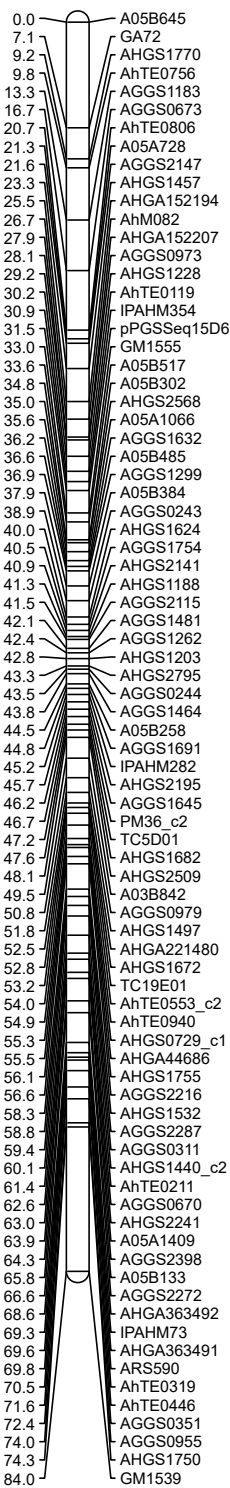

# LG17(B09)

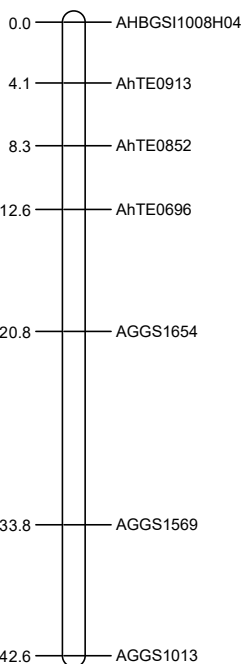

# LG18(B10)

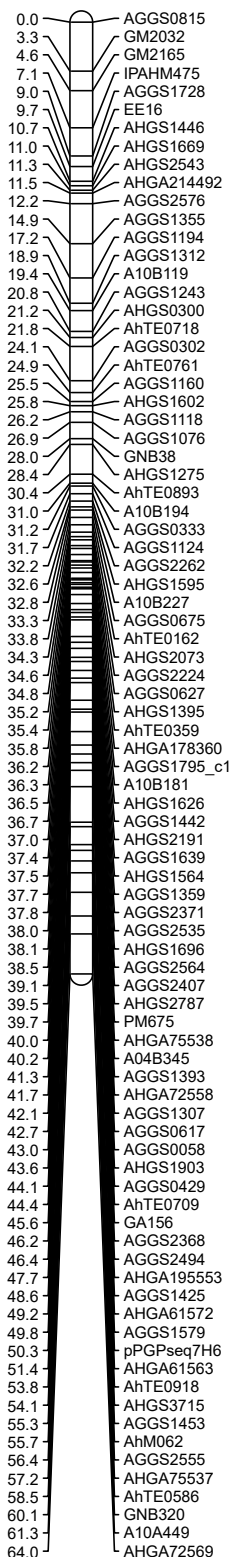

# LG19

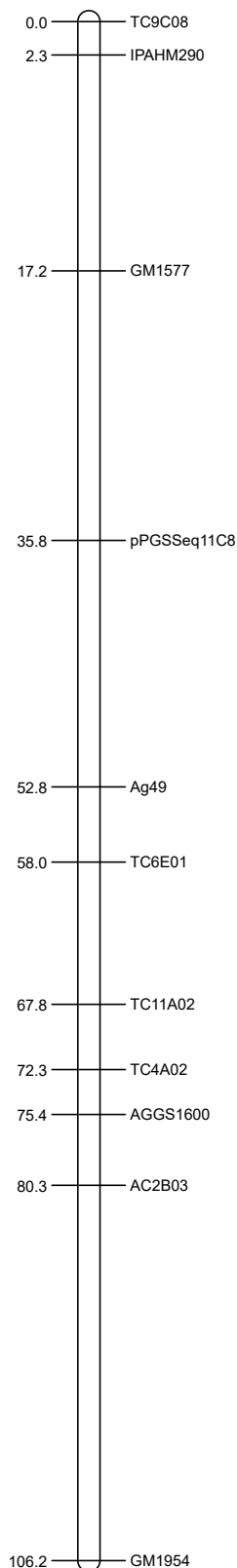

# LG20

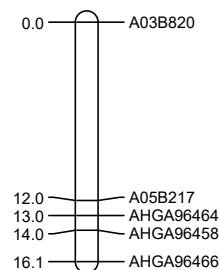

LG21

LG22

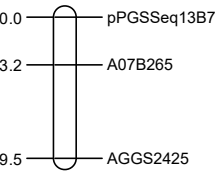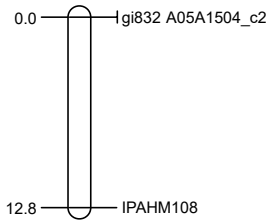

Supplement: Additional file 3: Figure S1. — Graphical presentation of genetic linkage map constructed in this study. (PDF 207 kb) [file 12864_2016_3456_MOESM3_ESM.pdf]

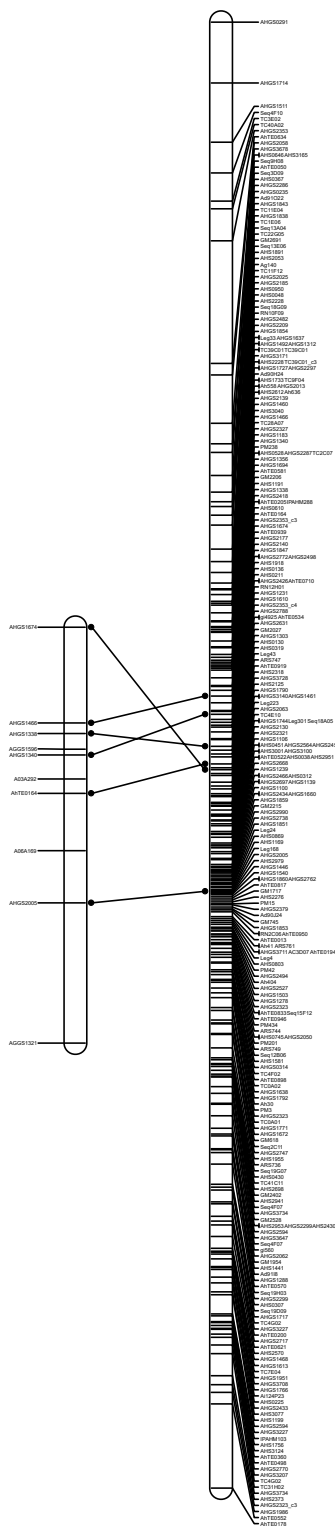

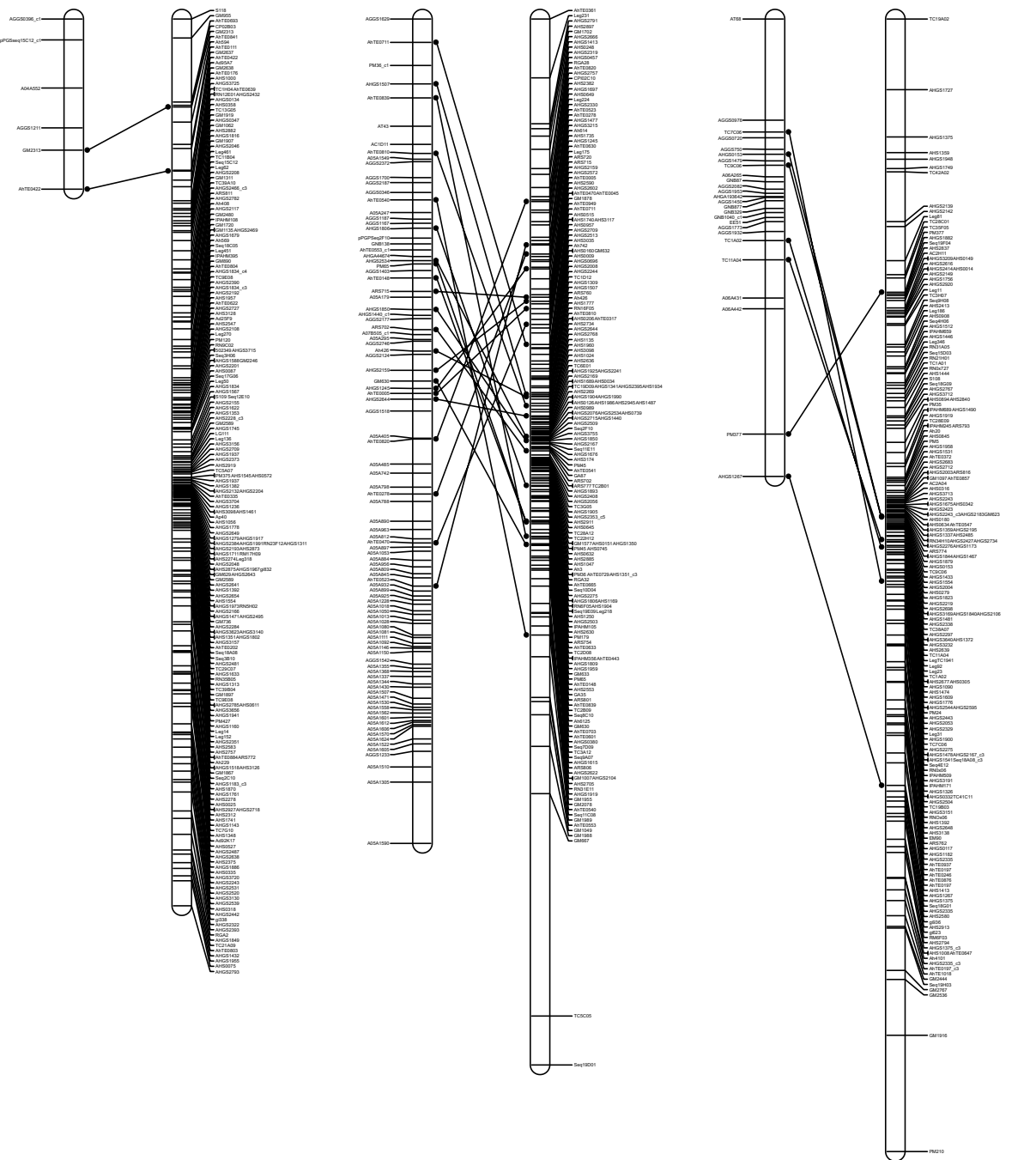

**LG09(A08)**

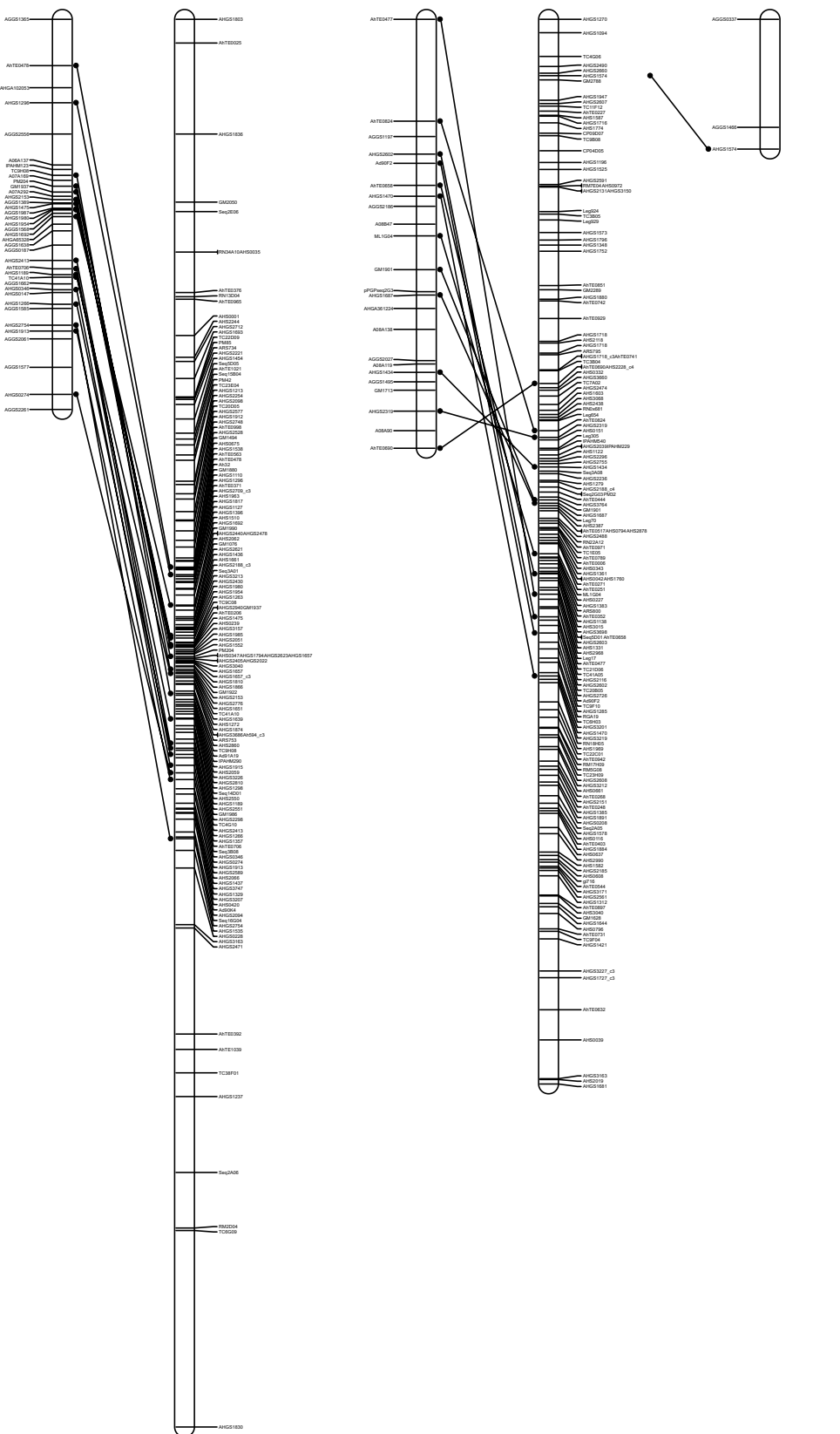

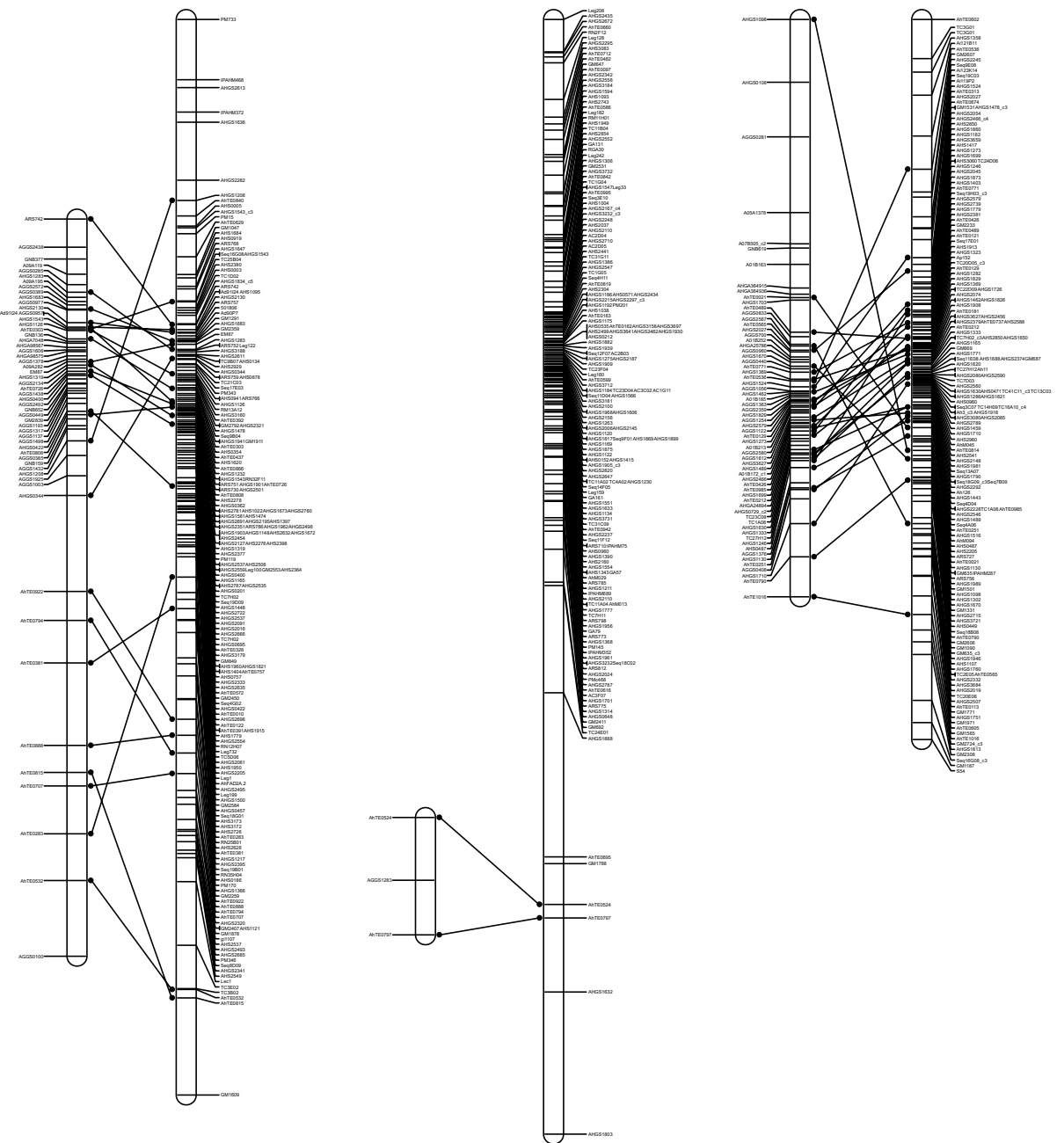

LG13(B02)

B02

LG14(B03)

B03

LG15(B04)

B04

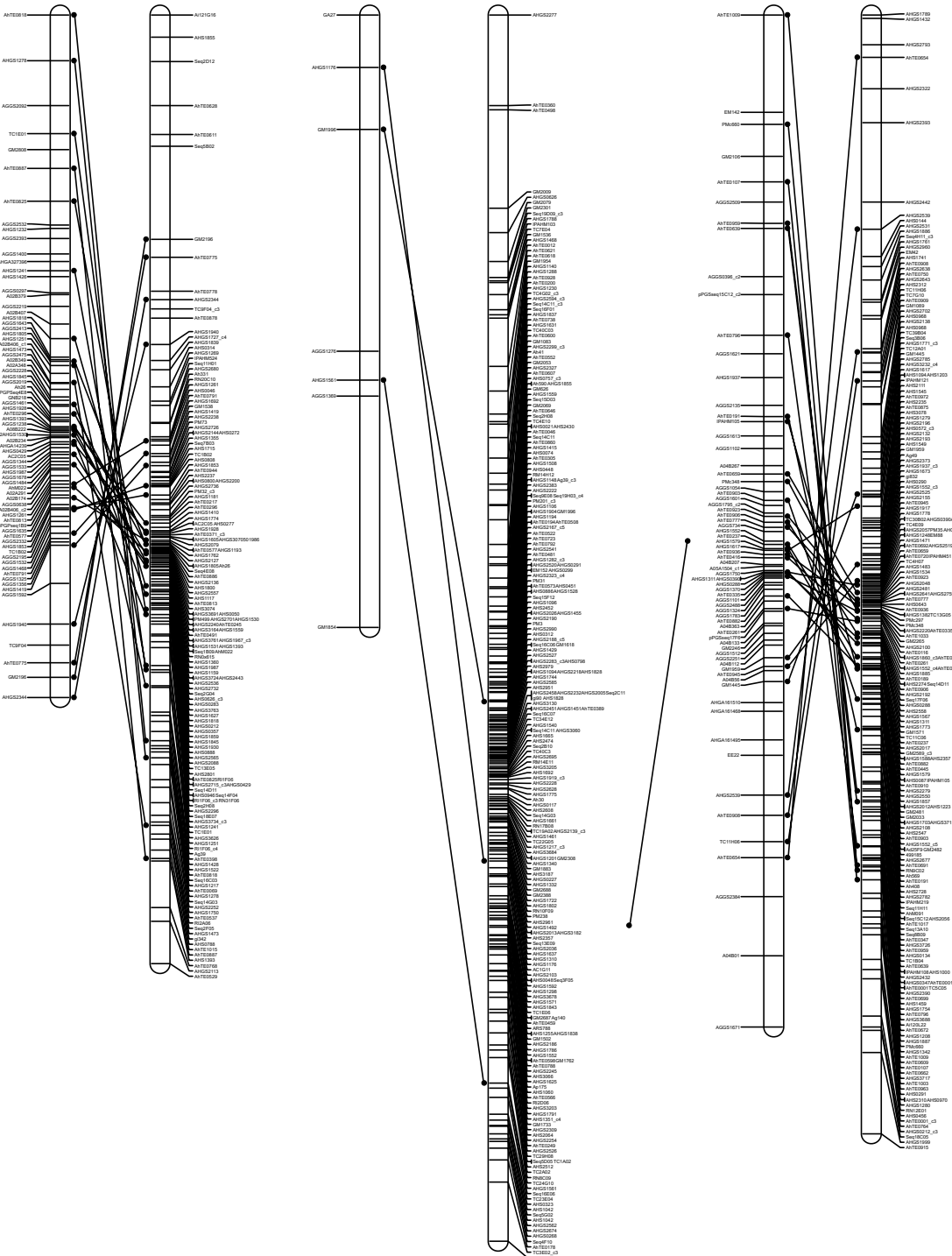

LG16(B05)

B05

LG17(B09)

B09

LG18(B10)

B10

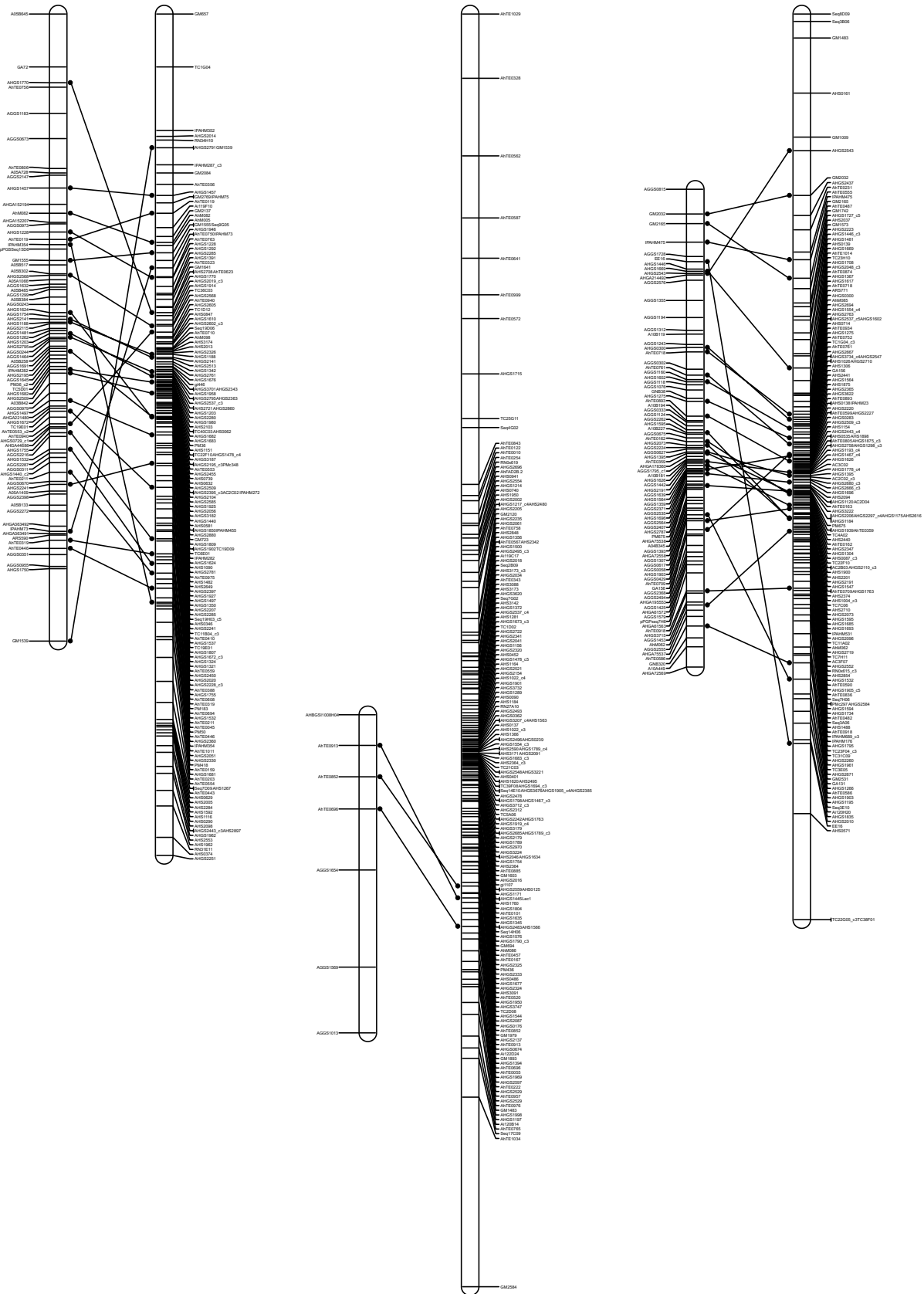

Supplement: Additional file 4: Figure S2. — Common markers between the newly constructed linkage map and the previously published integrated consensus map. (PDF 321 kb) [file 12864_2016_3456_MOESM4_ESM.pdf]

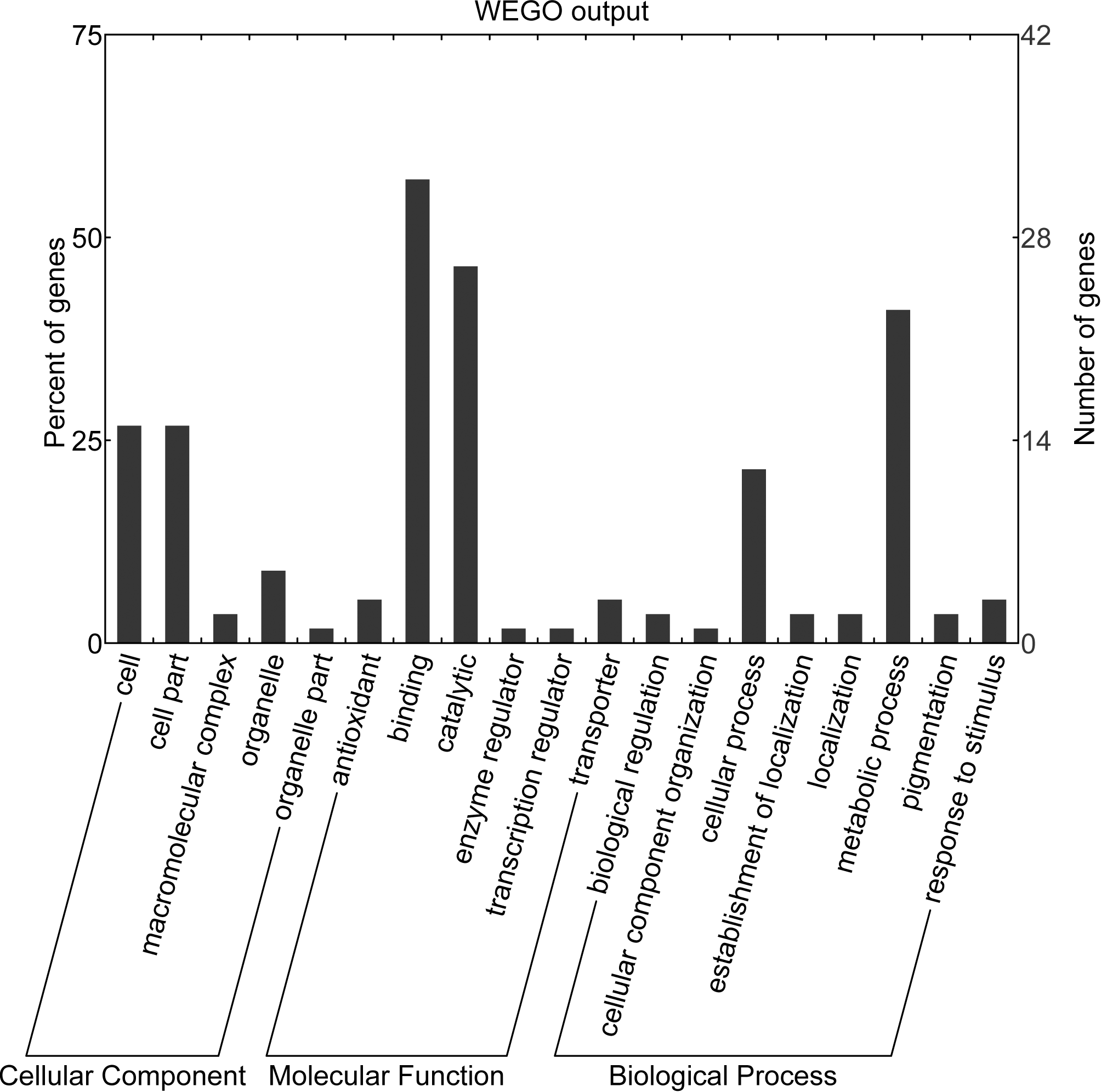

Supplement: Additional file 7: Figure S3. — GO annotations of genes in the dominant and co-localized interval on chromosome A05. (TIF 597 kb) [file 12864_2016_3456_MOESM7_ESM.tif]
